# Monitoring casbene synthase in *Jatropha curcas* tissues using targeted proteomics

**DOI:** 10.1186/s13007-021-00716-7

**Published:** 2021-02-06

**Authors:** Natália Pinto de Almeida, Domingos Ferreira Mélo Neto, Gabriel Reis Alves Carneiro, Andreza Raquel Barbosa de Farias, Gilberto Barbosa Domont, Francisco de Assis de Paiva Campos, Fábio César Sousa Nogueira

**Affiliations:** 1 Laboratory of Proteomics/LADETEC, Universidade Federal Do Rio de Janeiro, Rio de Janeiro, Brazil; 2 Proteomics Unit, Institute of Chemistry, Universidade Federal Do Rio de Janeiro, Rio de Janeiro, Brazil; 3 Department of Agricultural Sciences, Universidade Federal Do Ceará, Fortaleza, 60356-900 Brazil; 4 Department of Biochemistry and Molecular Biology, Universidade Federal Do Ceará, Fortaleza, Brazil

**Keywords:** Biofuel crops, Secondary metabolism, Phorbol esters, Mass spectrometry, Targeted proteomics, Selected reaction monitoring, Parallel reaction monitoring, Proteotypic peptide

## Abstract

**Background:**

Casbene synthase (CS) is responsible for the first committed step in the biosynthesis of phorbol esters (PE) in the Euphorbiaceae. PE are abundant in the seeds of the biofuel crop *Jatropha curcas* and its toxicity precludes the use of the protein-rich cake obtained after oil extraction as an animal feed and the toxicity of the fumes derived from burning PE containing biofuel is also a matter of concern. This toxicity is a major hindrance to exploit the potential of this crop as a source of raw material to produce biodiesel. For this reason, the current research on *J. curcas* is mainly focused on the understanding of the biosynthesis and site of synthesis of PE, as an avenue for the development of genotypes unable to synthesize PE in its seeds.

**Results:**

Here, we present targeted proteomics assays (SRM and PRM) to detect and quantify CS in leaves, endosperm, and roots of two *J. curcas* genotypes with contrasting levels of PE. These assays were based on the use of reference isotopic labeled synthetic peptides (ILSP) predicted from 12 gene models of CS from the *J*. *curcas* genome.

**Conclusion:**

Our targeted proteomics methods were able to detect and quantify, for the first time, CS gene products and demonstrate the distribution of CS isoforms only in roots from *J. curcas* genotypes with a high and low concentration of PE. These methods can be expanded to monitor CS, at the protein level, in different tissues and genotypes of *J. curcas*.

## Background

The suitability of *Jatropha curcas* seeds as a source of raw material for the production of biodiesel is grounded in its high potential yield (3.2 to 4.1 t seeds/ha), in the excellent technological properties conferred to the biodiesel by its fatty acid composition, and in the high content of fatty acids (30–45%) and protein (30–40%), which makes the seeds not only a good source of fatty acids but also of protein for animal feed [1]. As efforts to domesticate this species is reaching considerable success[2], the major hurdles preventing the exploitation in full of this potential are the high content of a class of toxic diterpenes, the phorbol esters, in the oil and the seed cake resulting from oil extraction, precluding the use of the protein-rich seed cake as feed for farm animals [3, 4]. Another major drawback is the lack of toxicological studies regarding the effects of the burning of phorbol esters containing biodiesel in closed quarters and the environment [5].

The phorbol esters (PE) are tetracyclic diterpenes made up of a tigliane (12-hydroxy-16-deoxyphorbol) as its basic structure and fatty acids esterified at C13 and C16 positions [6]. The toxicity of the PE, from which at least six kinds were identified in the seeds of *J. curcas*, is derived from its ability to act as diacylglycerol (DAG) analogs, activating protein kinase C, leading to the acceleration of cellular proliferation process, conferring to the PE a major role in tumor development [7, 8].

The biosynthetic pathway of PE is largely unknown, and in seeds, they are found in higher concentrations in the tegmen and endosperm and smaller amounts in leaves and roots [8]. Geranyl geranyl diphosphate (GGDP) is a key intermediary for the biosynthesis of diterpenoids, such as PE, taxol, gibberellins, etc. The conversion of GGDP to casbene by casbene synthase (CS) is believed to be a key step in PE biosynthesis [9]. Casbene synthase was first studied in the castor plant (*Ricinus communis*) [10], where it catalyses the synthesis of the phytoalexin casbene from GGDP; ensuing studies [6, 11] led to the cloning of a *J. curcas* CS gene (JcCSH) coding for a protein with high sequence similarity with the CS from *R. communis*. The *J. curcas* protein was localized in the chloroplast and displayed the motif DDXXD which is conserved among several terpene cyclases. Subsequently, it was shown that knockdown of two CS genes (*JcCASA163* and *JcCASD168*) decreased significantly the concentration of PE, thus adding weight to the hypothesis that CS is a key enzyme for the PE synthetic pathway [12]. Between ten and fourteen CS gene homologs were to be found in the *J. curcas* genome [13]. King et al. [9] characterized a physical cluster of diterpenoid genes, including CS and cytochrome P450s from the CYP726A subfamily, presenting evidence for the transcriptional co-regulation within this cluster. Later, Ha et al. [14] determined this physical gene cluster is located on chromosome 3.

Several proteome studies on *J. curcas* have been published on tissues such as the inner integument of seeds [15], embryos [16], endosperm [16–19], germinating seeds [16], seed organelles [20–23], and stem latex [24]. Although together these studies identified more than 4000 proteins, no CS was identified, and these studies did not attempt to find out whether the lack of CS identification was related to the dynamic range of the protein extracts under analysis. Only recently, Farias et al. (2020) gathered evidence for the presence of CS in the roots of two genotypes displaying contrasting levels of PE [25]. On the other hand, the literature abound on reports on the transcription of the CS gene in developing seeds [26, 27], mature seeds [26, 27], developing fruit [11, 14, 27], mature fruits [14], developing leaves [12, 26, 27], mature leaves [9, 26, 27]; roots [9, 26], flowers [9, 26], tegmen [12], and stem [9]. Unfortunately, none of these results were validated by protein targeted methods and may simply highlight the frequently found poor transcript-protein correlation [28]. These conflicting results emphasize the need for reliable protocols for CS identification and quantification. For this purpose, we present here a targeted proteomics strategy, namely selected reaction monitoring (SRM) and parallel reaction monitoring (PRM), to investigate the presence and abundance of CS in leaves, roots, and endosperm of two *J. curcas* genotypes displaying contrasting levels of PE. This strategy will be a useful tool to investigate the presence and abundance of CS in different tissues and to validate the results of transcript level studies.

## Results

**Fig. 1:**
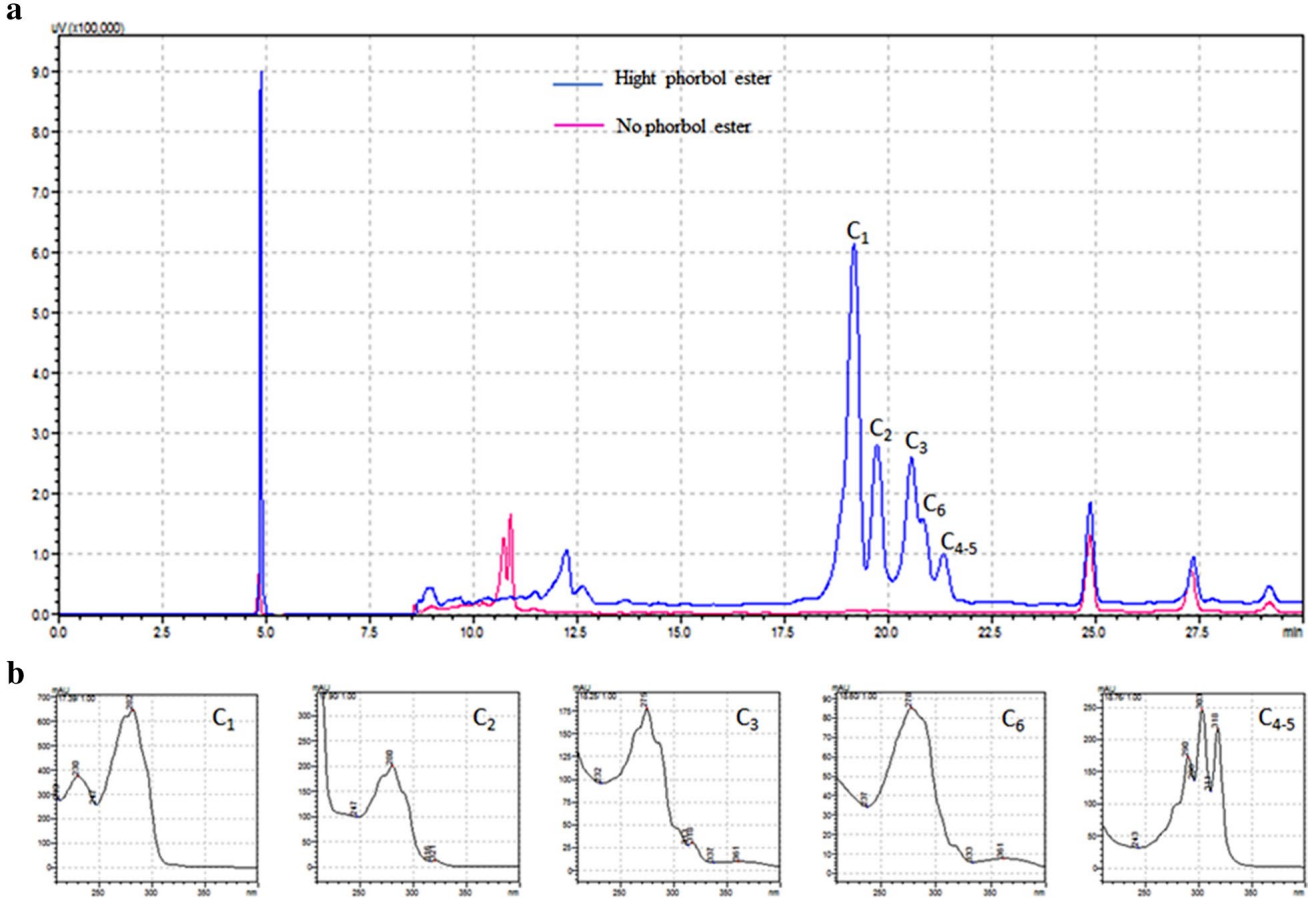
Phorbol esters seed content analysis by HPLC. **a** Chromatograms were obtained at 254 nm for the detection of phorbol esters in kernel seeds from two different genotypes of *J. curcas*. The blue and red lines represent the chromatograms from high and low PE level genotypes, respectively. The signals detected in 17.5–22.5 min corresponds to *Jatropha* factors C_1-6_. **b** UV light absorption spectra for each one of the *Jatropha* factors

**Fig. 2:**
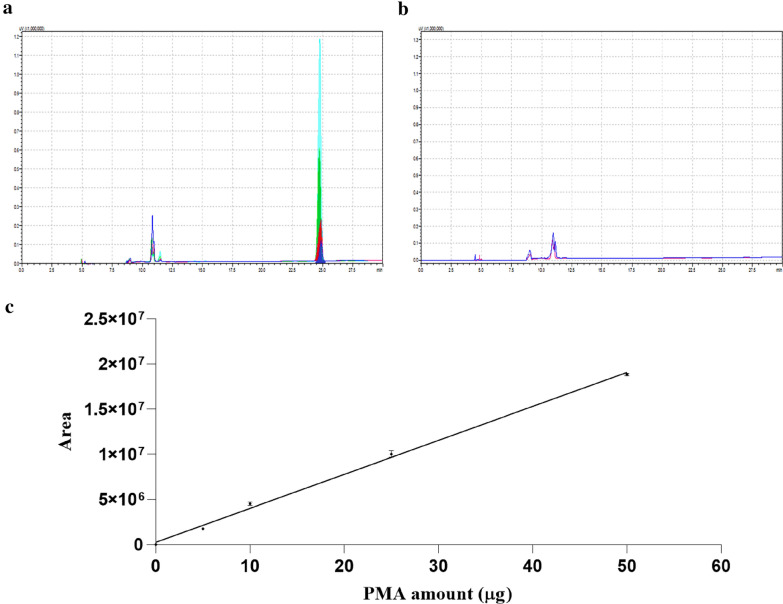
Phorbol 12-myristate 13-acetate (PMA) standard curve for phorbol ester quantification. **a** Chromatograms obtained at 254 nm for the detection of PMA standard. The light blue, green, red and dark blue lines represent the analysis of 50, 25, 10, 5 and 0 µg of PMA. **b** Chromatograms obtained at 254 nm for the detection of Ethanol anhydrous (TEDIA-High purity solvents), used as a blank run. **c** PMA standard curve. The y and x axes represent the mean of area under the curve ± SD and the amount of PMA standard analyzed, respectively. y = 376561x + 254,199; r^2^ = 0.9971

The concentration of PE in seeds in the genotype with high levels of PE in the seed (hereafter called HPE) was 0.5519 ± -0.0627 μg/mg seed kernel, while the levels in the low levels of PE (hereafter called LPE) genotype was below the detection level for this compound in the assay used. The chromatogram of seed extracts from the HPE genotype is shown in Fig. 1, where the presence of five peaks between 17.5–22.5 min. are shown, while seed extracts of the HPE genotype did not show any detectable signal in the same retention time window, thus confirming the contrasting levels of PE in these two genotypes. The standard curve used for quantification and the chromatograms obtained for PMA (Phorbol 12-myristate 13-acetate) detection are shown in Fig. 2.

As shown in Additional file 1: Table S1, 12 CS gene models were selected from the updated version of the *J. curcas* genome (JAT_r4.5) [13]. To avert the possibility that these gene models code for proteins other than CS, a BLASTp analysis against *J. curcas* entries from UniProt, NCBI, and the genome published in 2018 by Ha et al*.* [14] was carried out. The best hits for Jcr4S01081.10, Jcr4S01081.20 and Jcr4S01081.30 are CS1/JcCASA163/JcCSH, CS2 and JcCASA163/JcCSH, respectively [9, 11, 12]. Peptides EVELLNEK, LGVSYHFEEEIEENLTK, and IFNIQPTFLNEK that could be found in the first version of the Jatropha database [29], but not on any gene model from Jatropha Genome Database JAT_r4.5 [13], are tryptic peptides from casbene synthase 2 [9]. The best hit for the gene model Jcr4S03333.20 has sequence identity with a CS fragment deposited on UniProt (Access Number: A0A0B4KE70) and NCBI (Access Number: AFQ98016.1). This CS fragment has 94.5% of identity with JcCASD168 [12]. The identity of the products of gene models Jcr4S01081.10, Jcr4S01081.20, and Jcr4S01081.30 as casbene synthase was validated by expression of the relevant cDNAs in bacteria followed by the experimental demonstration of in vitro casbene synthase activity [9, 11].

In the case of the remaining gene models for which no transcriptional evidence is available, the BLASTp hits from the UniProt database were E6NUA6 (Jcr4S03853.10, Jcr4S06058.10, and Jcr4S01881.20), E6NUA8 (Jcr4S24303.10 and Jcr4S04482.10), E6NUA9 (Jcr4S04129.10) and E6NUB0 (Jcr4S03286.10 and Jcr4S17930.10). The above sequences correspond to homologs of the CS gene from *Ricinus communis*, as described in the first version of *J. curcas* genome database Jc_r3.0 [29].

Interestingly, peptide NLIQNIELIDTLR that we first identified as a tryptic peptide of a CS synthase in the first version of the Jatropha genome, can no longer be found on any gene model in the Jatropha Genome Database JAT_r4.5. However, a BLASTp search of this sequence against NCBI and Uniprot databases assign it to proteins from the terpene synthase family.

The list of targets and isotopically labeled synthetic peptides (ILSP) is shown in Additional file 2: Table S2, together with the optimized parameters for SRM, including the best transitions, the optimal collision energy, and the RF lens parameter for each target peptide and the ILSP. Likewise, Additional file 3: Table S3 shows the charge state and optimized CE for each precursor for the PRM analysis of CS. For PRM analysis, the injection time was adjusted and set up as 100 ms, and a scheduled method with a 4 min window was developed to guarantee more than eight acquisitions throughout a peak. For SRM, a scheduled method was used with a 4 min window also to guarantee a dwell time of 10 ms and more than eight acquisitions throughout a peak. All the ILSP were detected when analyzed only in 0.1% formic acid aqueous solution. Their detection was also tested when added to the samples, but it was not possible to identify some of them, presumably due to the matrix effect. For that reason, these ILSP were excluded in the final method. In Table 1, we describe the peptides monitored in the methods and the respective final concentration in the sample.

**Table 1:** Concentration of each ILSP in the *J. curcas* samples for CS analysis. Amino acids K and R are highlighted to represent isotopic labeling

CS gene models (Accession number JAT_r4.5*)	Peptide	Concentration (fmol/µL)		
		Root	Leaf	Endosperm
Jcr4S01081.30	GTEALEWL**K**	21.25	212.5	212.5
Jcr4S01081.30	LVNDITSHETEQD**R**	212.5	212.5	212.5
Jcr4S01081.20	FASFSLGN**R**	21.25	212.5	212.5
–	EVELLNE**K**	212.5	212.5	212.5
–	IFNIQPTFLNE**K**	21.25	212.5	212.5
Jcr4S01081.20	HIGSALEQPVH**K**	–	–	212.5
Jcr4S01081.20	EAYQELV**K**	21.25	212.5	212.5
Jcr4S01081.20	GTEAFEWLK***	21.25	212.5	212.5
Jcr4S01081.10	LNEQLIV**R**	21.25	212.5	212.5
Jcr4S01081.10	YGDGYTDSSQLK**	21.25	212.5	212.5
Jcr4S03333.20	LEILAAQSSPHLANR***	10.62	212.5	212.5
Jcr4S03333.20	DLNLVEELPYVR**	21.25	212.5	212.5
Jcr4S03333.20	YSDSYTFPTIL**K**	21.25	212.5	212.5
Jcr4S03853.10	ALLNLFAETENDETEG**R**	212.5	212.5	212.5
Jcr4S03853.10	YSDSYTYSTILK**	212.5	212.5	212.5
Jcr4S04129.10	AVLDLFEETSNIGSK**	21.25	212.5	212.5
Jcr4S04129.10	LLNDVVSH**K**	–	212.5	212.5
Jcr4S04482.10	NALIFSFQ**K**	21.25	212.5	212.5
Jcr4S04482.10/ Jcr4S24303.10	QYISVYEEDES**R**	212.5	212.5	212.5
Jcr4S06058.10	GEDVLDEAFAFA**K**	212.5	212.5	212.5
Jcr4S06058.10/ Jcr4S01881.20	NILTWPFQR**	10.62	212.5	212.5
Jcr4S06058.10/ Jcr4S01881.20	QELALLS**R**	21.25	212.5	212.5
–	NLIQNIELIDTLR**	21.25	212.5	212.5
Jcr4S03286.10/ Jcr4S17930.10	GNDGYTNPSSL**K**	212.5	212.5	212.5

^*^ Available in https://www.kazusa.or.jp/Jatropha/

^**^ Endogenous targeted peptides identified in SRM and PRM analysis

^***^ Endogenous targeted peptides identified in SRM analysis

For the SRM and PRM assays, we set as the internal positive control for the root, endosperm, and leaf samples, the proteins peptidyl-prolyl cis–trans isomerase (A0A067LPN3), legumin type B (A0A067K3Z1) and the chloroplast photosystem II 10 kDa (D6BRD6), respectively, by the reason that these proteins were well identified in previous shotgun proteomics experiments [25]. Additional file 4: Table S4 shows SRM and PRM transitions to be monitored.

**Fig. 3:**
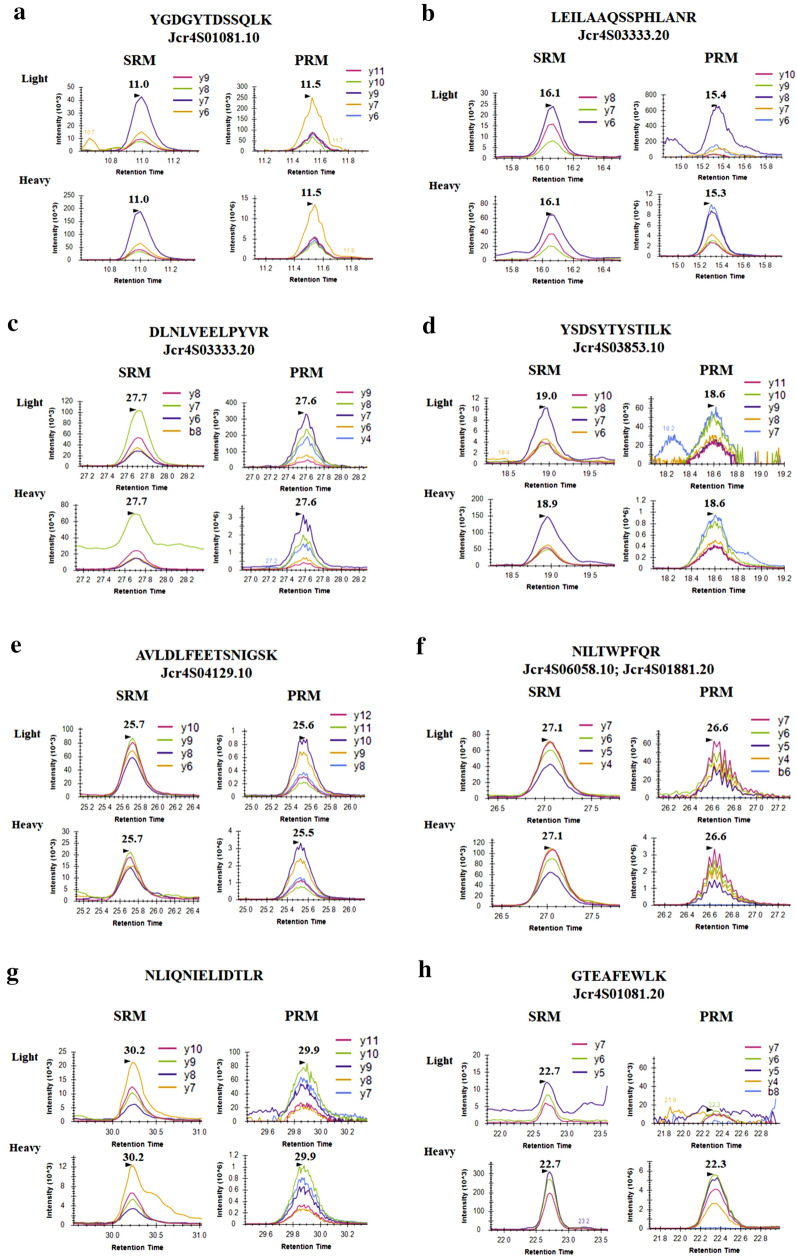
Results from the casbene synthase analysis of roots samples from *J. curcas*. XIC of fragment ions from heavy and endogenous peptides identified in the SRM and PRM analysis of casbene synthase. All the peptides identified showed rdotp value around 1. Figure 2a-h represents the results for each peptide identified. **a** YGDGYTDSSQLK (Jcr4S01081.10). **b** LEILAAQSSPHLANR (Jcr4S03333.20). **c** DLNLVEELPYVR (Jcr4S03333.20). **d** YSDSYTYSTILK (Jcr4S03853.10). **e** AVLDLFEETSNIGSK (Jcr4S04129.10). **f** NILTWPFQR (Jcr4S06058.10 e Jcr4S01881.20). **g** NLIQNIELIDTLR. **h** GTEAFEWLK (Jcr4S01081.20)

**Fig. 4:**
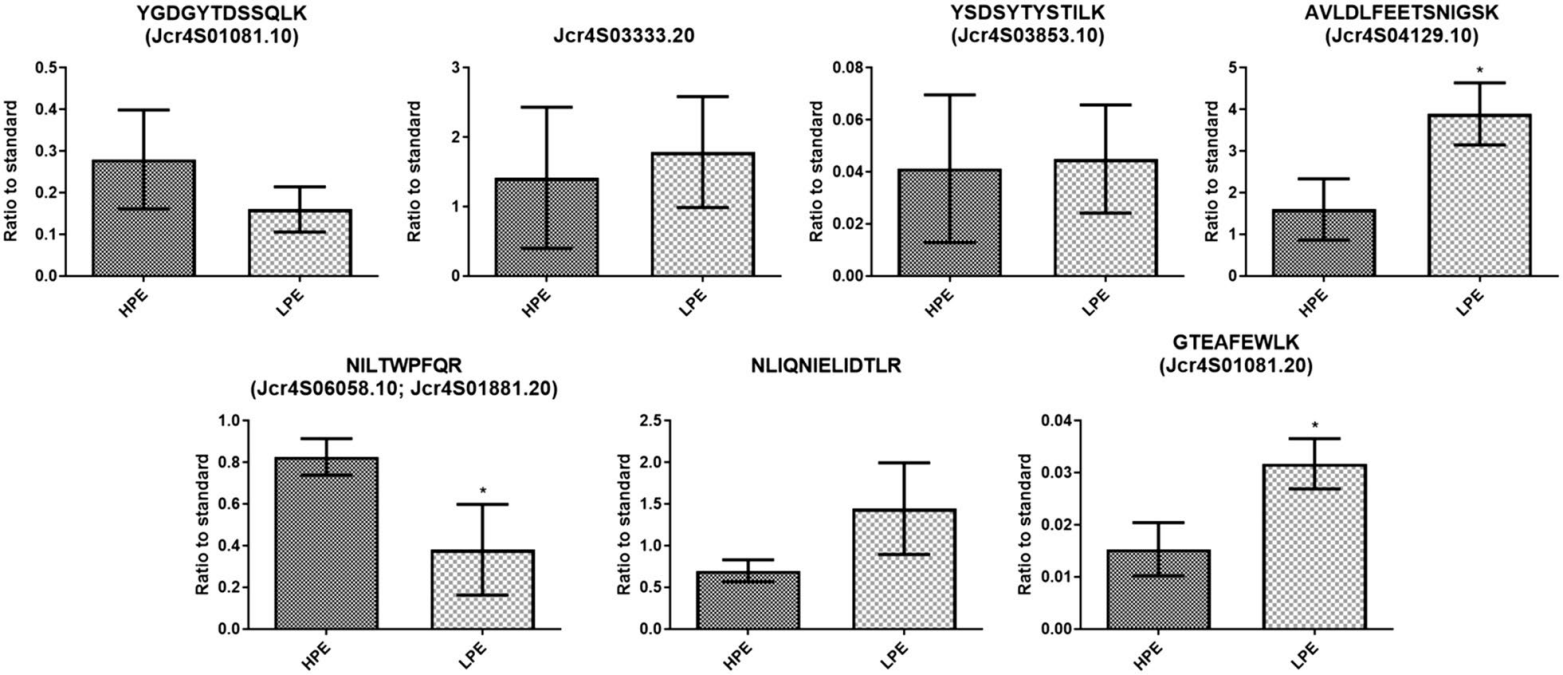
Casbene synthase relative quantification results for the SRM analysis of root samples. Relative quantification of endogenous peptides identified in roots (n = 3). The chromatographic areas of the most intense fragment from the endogenous peptide showing no signal interference were normalized by the area of the same fragment from its respective heavy peptide. For proteins identified from more than two peptides, the results are expressed as the sum of the relative quantification of each peptide. The results are shown by graphic bars representing the mean ± SD. T-test p-value are shown: * p-value < 0.05

**Fig. 5:**
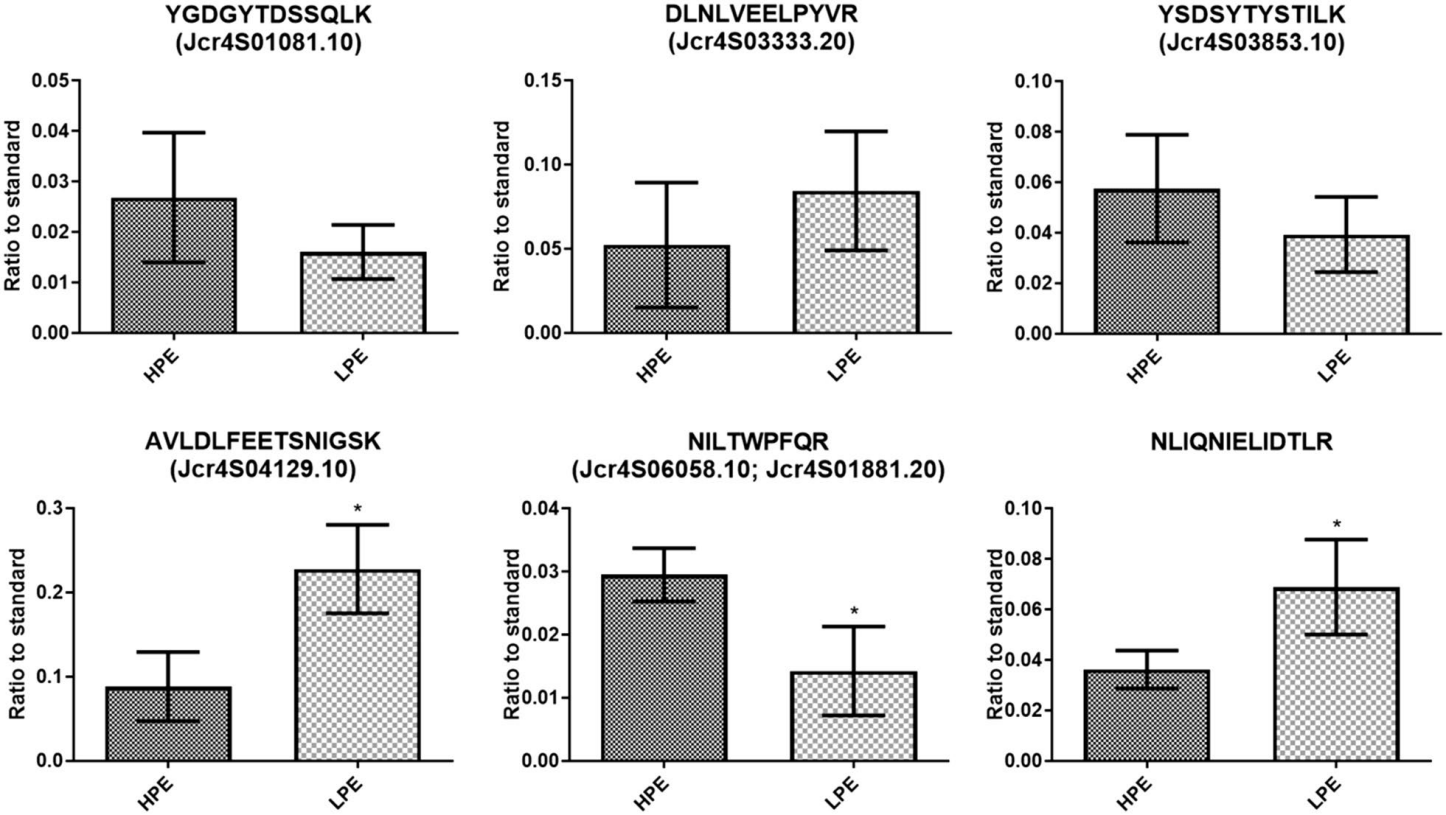
Casbene synthase relative quantification results for the PRM analysis of root samples. Relative quantification of endogenous peptides identified in roots (n = 3). The chromatographic areas of the most intense fragment from the endogenous peptide showing no signal interference were normalized by the area of the same fragment from its respective heavy peptide. For proteins identified from more than two peptides, the results are expressed as the sum of the relative quantification of each peptide. The results are shown by graphic bars representing the mean ± SD. T-test p-value are shown: * p-value < 0.05

**Fig. 6:**
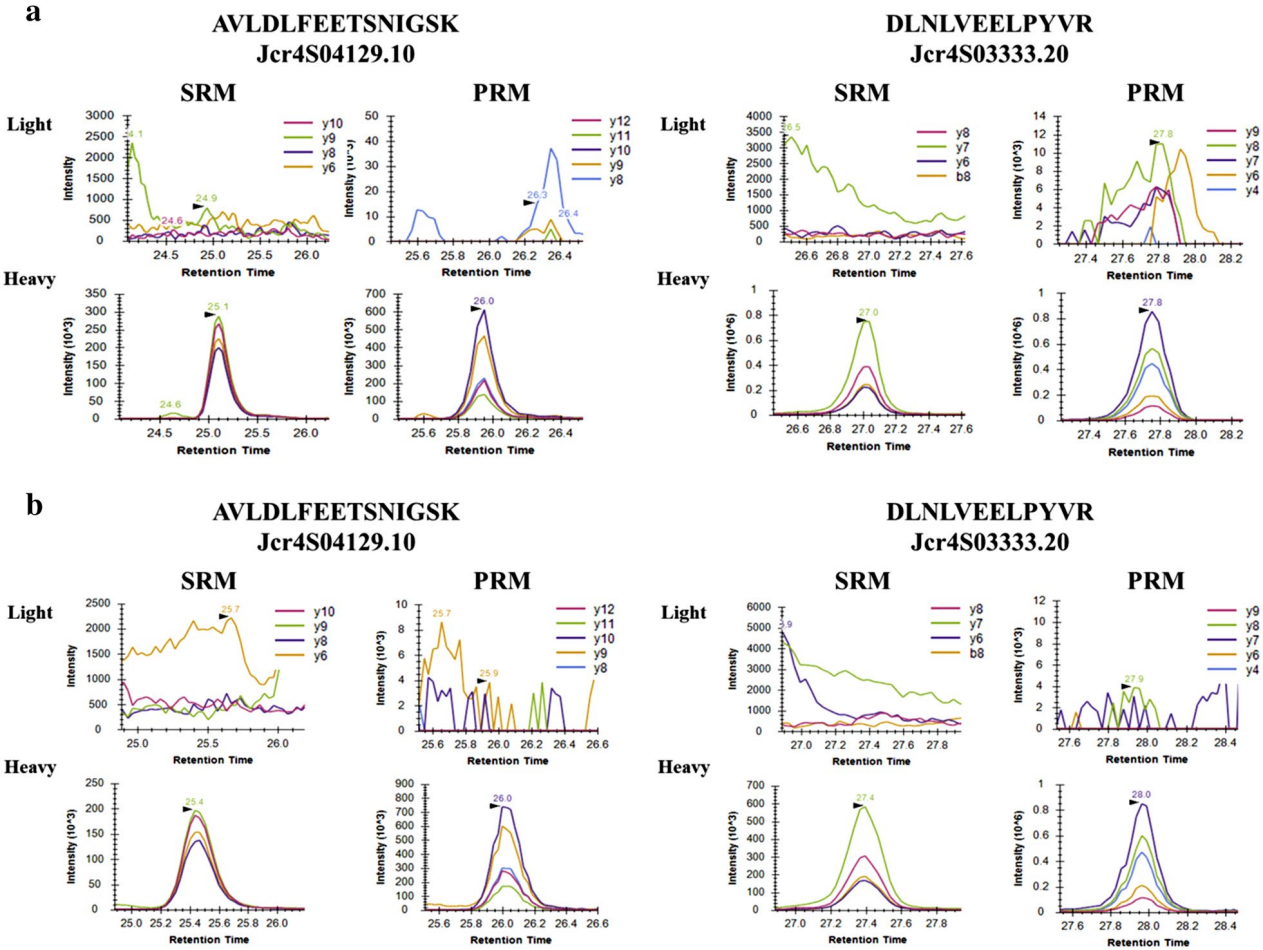
Results from casbene synthase analysis of leaf and endosperm samples from *J. curcas*. XIC of fragment ions from heavy and endogenous peptides monitored in the SRM and PRM analysis. Figure 3a, b represent the results for the analysis of peptides AVLDLFEETSNIGSK (Jcr4S04129.10) and DLNLVEELPYVR (Jcr4S03333.20) in endosperm and leaf, respectively

The use of the SRM assay allowed for the identification of eight endogenous peptides (Fig. 3) in the roots of the HPE and LPE genotypes and, consequently, relative quantification (Fig. 4) of seven CS gene models in addition to the peptide NLIQNIELIDTLR. PRM analyses confirmed the presence and quantification of six CS target peptides (Figs. 3, 5). However, when applied SRM and PRM methods to leaves and endosperm, no endogenous peptides were identified in the corresponding samples (Fig. 6). The rdotp parameter calculated by Skyline software was evaluated for the peptides identified in each of the biological and technical replicates, resulting in values around 1.0, although the values below 1.0 can be explained by the fact that the endogenous peptide peak is very close to the noise or there is interference (Additional file 5: Table S5).

For the SRM analysis, no statistical differences in the relative concentration of three CS gene models and peptide NLIQNIELIDTLR in the two contrasting genotypes were observed (Fig. 4 and Table 2). However, the relative abundances of peptides GTEAFEWLK (Jcr4S01081.20), AVLDLFEETSNIGSK (Jcr4S04129.10) e NILTWPFQR (Jcr4S06058.10 and Jcr4S01881.20) are significantly different between the two contrasting genotypes (Table 2). The relative levels of peptides GTEAFEWLK (Jcr4S01081.20) and AVLDLFEETSNIGSK (Jcr4S04129.10) is 2.07 and 2.43 times higher in the LPE genotype, respectively, while peptide NILTWPFQR (Jcr4S06058.10 and Jcr4S01881.20) is 2.17 times more abundant in the high PE levels genotype.

**Table 2:** Relative quantification of casbene synthase candidates identified by analysis of *J. curcas* root samples by the SRM method (N = 3)

Targets	Mean ± SD		LPE/HPE	p-valor
	HPE	LPE		
Jcr4S01081.10	0.2800 ± 0.1188	0.1602 ± 0.05442	0.5721	0.1875
Jcr4S03333.20	1.415 ± 1.015	1.786 ± 0.7982	1.2622	0.645
Jcr4S03853.10	0.04124 ± 0.02836	0.04493 ± 0.02074	1.0895	0.8644
Jcr4S04129.10	1.601 ± 0.7345	3.893 ± 0.7459	2.4316	0.0192
Jcr4S06058.10/ Jcr4S01881.20	0.8266 ± 0.08748	0.3806 ± 0.2180	0.4604	0.0302
NLIQNIELIDTLR	0.6998 ± 0.1311	1.445 ± 0.5501	2.0649	0.0846
Jcr4S01081.20	0.01532 ± 0.005107	0.03173 ± 0.004812	2.0711	0.0155

In the PRM assay (Fig. 5 and Table 3), the relative abundance of peptide AVLDLFEETSNIGSK (Jcr4S04129.10) is 2.58 times higher in the LPE genotype, while the relative abundance of peptide NILTWPFQR (Jcr4S06058.10, Jcr4S01881.20) is 2.07 times higher in the HPE genotype, which is in line with the quantification results obtained for these peptides in the SRM experiments. Conversely, the relative abundance of peptide NLIQNIELIDTLR showed a significant difference in abundance between the two genotypes, being 1.90 times more abundant in the LPE genotype.

**Table 3:** Relative quantification of casbene synthase candidates identified by analysis of *J. curcas* root samples by the PRM method (N = 3)

Targets	Mean ± SD		LPE/HPE	p-valor
	HPE	LPE		
Jcr4S01081.10	0.02686 ± 0.01287	0.01605 ± 0.005377	0.5975	0.2507
Jcr4S03333.20	0.05226 ± 0.03720	0.08438 ± 0.03537	1.6146	0.3394
Jcr4S03853.10	0.05755 ± 0.02132	0.03933 ± 0.01490	0.6834	0.2917
Jcr4S04129.10	0.08816 ± 0.04097	0.2277 ± 0.05261	2.5828	0.0223
Jcr4S06058.10/ Jcr4S01881.20	0.0295 ± 0.004220	0.01425 ± 0.007033	0.483	0.0322
NLIQNIELIDTLR	0.03622 ± 0.007513	0.06884 ± 0.01883	1.9006	0.0495

## Discussion

The concentration of PE in the seed kernels was 0.5519 μg/mg, corresponding to at least six PE types. The diversity and concentration of PE in the HPE genotype are in line with results obtained from the analysis of other *J. curcas* genotypes with toxic seeds [30–34]. We were unable to quantify PE in the LPE genotypes, despite the low detection limit of the method.

By using SRM and PRM assays, endogenous peptides corresponding to tryptic peptides of gene models Jcr4S01081.10, Jcr4S01081.20, Jcr4S03333.20, Jcr4S03853.10, Jcr4S04129.10, Jcr4S06058.10/Jcr4S01881.20, and the peptide NLIQNIELIDTLR were identified in roots, but not in leaves and endosperm of the HPE and LPE genotypes. Although the expression of CS transcripts in different tissues and developmental stages of *J. curcas* have been reported [9, 11, 12, 14, 26, 27], no corresponding data on the proteins encoded by the transcripts were produced. Only recently a single proteomics study leads to the identification of CS in roots of *J. curcas* [25]. Together these results draw attention to the lack of correlation between experiments at the transcript and protein levels and the use of targeted proteomics approaches to validate gene transcription studies. The fact remains that CS is deposited in the roots of genotypes with no or very low levels of PE which raises the possibility that the absence of PE in seeds of LPE may be the result of other regulation in the CS leading to its inactivation.

Gene JcCASA163 studied by Nakano et al. [11], corresponds to a CS which was the source of one peptide here identified (YGDGYTDSSQLK). Transcription of this gene occurs in young leaf, inner skin, and seeds at six developmental stages. However, the results from our targeted proteomics analysis by SRM and PRM indicated the presence of the product of gene JcCASA163 in roots of the HPE and LPE genotypes, but not in leaves or seeds. The absence of CS in these tissues of the contrasting genotypes gives weight to a suggestion made previously by the literature [11, 15, 18, 21] that the PE or its intermediary metabolites are synthesized in roots and subsequently translocated to the shoot.

Li et al. in 2015 observed that silencing CS genes *JcCASA163* and *JcCASD168* resulted in the reduction of PE accumulation in *J. curcas* seeds. This was the first evidence that this step could be important for the regulation of PE biosynthesis [12]. Therefore, we were interested to evaluate if the abundance of CS enzymes is related to the regulation of this pathway. Here, we monitored the identification of peptides from the CS gene model Jcr4S01081.10, which shows high similarity with *JcCASA163*. The quantification of peptide YGDGYTDSSQLK did not show any difference in abundance between the two genotypes. Our result indicates that the abundance of this CS enzyme isoform is not related to the regulation PE biosynthesis pathway. These results show the relevance to evaluate the gene expression assays using protein level data.

In SRM assays, the HPE showed higher levels of the proteins encoded by gene models Jcr4S06058.10 and Jcr4S01881.20, while the LPE genotype showed higher abundance for gene models JcR4S04129.10 and Jcr4S01081.20. The PRM assays confirmed the higher relative abundance in the LPE genotype of the protein coded by gene model JcR4S04129.10. The PRM assay also indicated the higher relative abundance of the putative terpene synthase in the LPE genotype. The observation that the proteins coded by gene models Jcr4S04129.10 and Jcr4S01081.20 have higher abundance in LPE genotype may indicate the possibility that the abundance of these enzyme isoforms is not important for the regulation of PE accumulation in the seeds. The differential regulation of the activity/expression of the CS genes and its products merits further experimental approaches.

In this work, we identified three different isoforms of CS which can convert geranylgeranyl diphosphate into casbene [9, 11, 12], plus four other isoforms that have not been previously identified in *J. curcas* tissues either by RNA or protein studies. All these isoforms are present in roots of HPE and LPE genotypes but were not detected in leaves or seeds. These facts lead us to suggest that not all the CS isoforms are responsible for the low levels of phorbol esters in the LPE genotype, which is in line with the observation by King et al. [9] that the locus responsible for the loss of PE in seeds of some genotypes of *J. curcas* is in a separate linkage group from that of the gene cluster responsible for the first steps in the biosynthesis of diterpenoids.

## Conclusion

Targeted proteomics methods show higher selectivity, sensitivity, and wider linear dynamic range than shotgun proteomics, resulting in a more accurate quantitative result [35]. These methods are widely applied for validation of the results obtained by DDA methods and for the analysis of low abundance proteins [36–38]. As many previous proteomic works failed in detecting the CS enzyme in different tissues of *J. curcas*, we developed targeted methods for the detection and quantification of different CS isoforms. The targeted proteomics-based on SRM and PRM assays developed here were able for the first time to detect and quantify the CS gene products and demonstrate the distribution of CS isoforms in roots from HPE and LPE genotypes of *J. curcas*. We did not detect any CS isoform in the endosperm and leaves. These results may indicate a relevant role of roots in diterpene metabolism. We also have evidence that different CS isoforms can play different functions in the metabolism, and that not all of them have a relevant role in the accumulation of PE. Indeed, the hypotheses raised in the present paper still need to be validated to have a better understanding of PE accumulation.

## Methods

### Plant material and sample preparation

Voucher specimens of HPE and LPE genotypes are deposited at the Herbarium Prisco Bezerra, Federal University of Ceará, under numbers EAC62156 and EAC62157. The endosperm, leaves, and roots collection and preparation for protein extraction were performed as described [25].

### Phorbol ester extraction and content analysis

The mature seed kernel from the two genotypes of *J. curcas* was isolated, lyophilized, macerated, and stored at − 20 °C. The phorbol ester extraction was performed as described by He et al. [31] and King et al. [39], except for minor modifications. After kernel preparation, 2.2 mL of n-heptane/isopropanol solution (3:2) was added to 300 mg of this material, and the suspension was stirred for 1 h. The mixture was centrifuged (1000*g*, 35 min) and the supernatant was separated. Two more extractions were performed with 2.2 mL of n-heptane/isopropanol solution (3:2) and 1.5 mL of isopropanol. The supernatants were combined and dried in a thermostatic bath with nitrogen flow. The extract was dissolved in 1 mL of n-heptane, in which another 1 mL of acetonitrile was added. This mixture was stirred briefly and centrifuged (1000*g*, 35 min). The n-heptane phase was discarded, and the acetonitrile phase was dried. The sample was dissolved in 300 μL of acetonitrile.

After PE extraction, 100 μL of the sample was injected in Gemini C18 analytical column (Phenomenex; 250 × 4.6 mm; particle size 5.0 μm; 110 Å) and analyzed using a UFLC Shimadzu. The temperature was kept at 30 °C and the flow was 0.7 mL min^−1^. The elution was performed with a linear gradient of H_2_O, 0.1% TFA (phase A) and ACN, 0.1% TFA (phase B). The elution steps consisted of 80 to 90% of phase B for the first 10 min, followed by a step of 90 to 100% of phase B for 6 min and isocratic low with 100% of phase B for the last 16 min. The quantification was done by integrating the peaks referring to phorbol esters, followed by a comparison to a curve of the PMA standard (Phorbol 12-myristate 13-acetate, Sigma Chemical), built in the quantities of 0, 5, 10, 25, and 50 μg. The detection of phorbol esters and PMA were performed at 254 nm.

### Protein extraction

Protein extraction from each tissue is described in [25] and was performed according to Vasconcelos et al. [40]. Briefly, tissue powder was mixed with polyvinylpolypyrrolidone (PVPP) and pyridine buffer (50 mM pyridine, 10 mM Thiourea, 1% SDS, pH 5,0) in a proportion of 1:2:40 (w/w/v) and stirred for at least 4 h at 4 °C. The mixture was centrifuged (10,000 rpm, 4 °C), the supernatant was separated and the proteins were precipitated with 10% TCA/acetone overnight [40]. The pellet was washed with cold acetone and then dried at room temperature. This procedure was performed for three biological replicates.

### Sample digestion

Proteins were solubilized with 7 M urea/2 M thiourea/5 mM TEAB solution. The samples were quantified by Qubit Reagent (Qubit® Quantitation Kit – Invitrogen) and 80 µg of proteins were submitted to the digestion procedure. First, proteins were reduced with 10 mM DTT for 1 h at 30 °C and alkylated with 40 mM IAA, in the dark, for 30 min at room temperature. After this, the mixture was diluted 10× with 50 mM TEAB. The trypsin was added with a proportion of 1:50 w/w and the reaction was carried out for 18 h at 35 °C. The reaction was stopped with formic acid with a final concentration of 1%. Lastly, sample peptides were desalted using C18 Reverse Phase Chromatography Micro SpinColumns (Harvard Apparatus).

### Prediction of CS peptides and development of the targeted proteomics methods based on ILSP

Casbene synthase gene models were gathered from the Jatropha Genome Database (JAT_r4.5 version: https://www.kazusa.or.jp/Jatropha/), and further evaluated by search in the NCBI and Uniprot databases, together with experimental data from the literature. Skyline Software 4.2 was used to choose potential target peptides. The ILSP (SpikeTides™ L) were synthesized by JPT Peptide Technologies. The purity of the labeled amino acids is estimated in the range of 97%-99%. For the SRM strategy, the ILSP were optimized through direct infusion on TSQ Quantiva Triple Quadrupole (Thermo Scientific) to define the ideal parameters for the analysis of each peptide. The optimization was performed using the first quadrupole with a resolution of 0.7 or 0.4 (FWHM), CID gas pressure of 1.5 mTorr, and a collision energy (CE) range of 5 to 55 V with steps of 10 V. Three transitions were selected for each target peptide, cycle, and dwell time were optimized to have at least eight acquisitions for each target peptide and more than 10 ms for each transition analysis. Peptides were diluted in 0.1% formic acid aqueous solution and analyzed in the system EASYII-nanoLC coupled to the nESI-TSQ Quantiva to evaluate the detection of the transitions. For the PRM strategy, the ILSP mixture was analyzed on an EASY1000-nanoLC (Thermo Scientific) coupled to the nESI-QExactive Plus (Thermo Scientific) mass spectrometer, to find out the ideal CE and injection time.

Positive controls for SRM and PRM methods were based on previous *bottom-up* proteomic analysis of *J. curcas* tissues [15, 18, 20, 21, 25]. A spectral library from these data was built on Skyline 4.2 and the method for positive control was also developed on this software.

### Mass spectrometer analysis

The SRM and PRM CS analyses used an EASYII-nano LC coupled to the nESI-TSQ Quantiva and in the nESI-Q Exactive Plus mass spectrometer (Thermo Scientific), respectively. Two µg of tryptic peptides from each sample, spiked with the ILSP mixture, were loaded into a trap column C18 Acclaim PepMap 75 µm × 2 cm (Thermo Scientific) and fractioned in a C18 column PicoChip 75 µm × 105 mm (New Objective). In both methods, sample elution was performed using a gradient of 5 to 45% phase B (95% acetonitrile/0,1% formic acid) for 40 min, 45 to 95% for 10 min, 95% of phase B was maintained for 12 min, 95% to 5% in 3 min and 5% for the last 8 min. The running time was 73 min for each sample and the flow applied was 250 nL/min. For the SRM analysis, the ILSP mixture is described in Table 1. Regarding the PRM analysis, the concentration of each peptide in the ILSP mixture was 212.5 fmol/µL.

SRM parameters were: positive acquisition mode, the first and third quadrupoles were set up with a resolution of 0.7 (FWHM), precursor ions fragmentation carried out with Argon gas with a CID gas pressure of 1.5 mTorr. Regarding the ion source parameters, the spray voltage and the ion transfer tube temperature used was 2.6 kV and 280 °C, respectively, and the sweep gas was set up as 0.

PRM parameters were: positive acquisition mode, 17,500 (at m/z 200) orbitrap resolution, 5E5 AGC target, 2 m/z isolation window and 0.5 m/z offset. The ion source parameters as spray voltage, capillary temperature, and S-lens were set up as 2760 V, 250 °C, and 70, respectively.

### Data analysis

The analysis of SRM and PRM results were performed with Xcalibur v.2.2 and Skyline v. 19.1.193 software. Transitions relative intensities were evaluated by the rdotp values provided by the software Skyline v.19.1.0.193. In the case of PRM results, rdotp determination was performed using the five most intense transitions. Only transitions without signal interference were considered for the determination of rdotp. The relative intensity of the positive control transitions was evaluated by the dotp parameter.

The relative quantification was performed and normalized using the ratio between the chromatographic area of the most intense transition from each targeted endogenous peptide by its corresponded ILSP. The occurrence of signal interference was evaluated and fragments showing interference were not used. In that case, the next most intense was selected. The relative quantification of gene models identified by the presence of more than one peptide was inferred by the sum of normalized abundance for each peptide. After normalization, the ratio between HPE and LPE was calculated. For statistical analysis, we used t Student test on GraphPad Prism 6 software, considering statistically different the results with p-value < 0.05.

## Supplementary Information

**Additional file 1: Table S1.** Selection of casbene synthase candidates.

**Additional file 2: Table S2.** Optimized parameters for the SRM analysis of casbene synthase.

**Additional file 3: Table S3.** Optimized parameters for the PRM analysis of casbene synthase.

**Additional file 4: Table S4.** Optimized parameters for the SRM analysis of peptides from positive controls.

**Additional file 5: Table S5.** SRM and PRM result from casbene synthase analysis.

## Data Availability

The SRM and PRM data files (ProteomeXchange Id: PXD019525) have been deposited at PRIDE—Proteomics Identification Database.

## Abbreviations

PE: Phorbol ester
MS: Mass spectrometry
SRM: Selected reaction monitoring
PRM: Parallel reaction monitoring
CS: Casbene synthase
rdotp: Ratio dot product
dotp: Dot product
HPE: High phorbol ester levels
LPE: Low phorbol ester level
